# Molecular docking analysis of compounds from Justica adhatoda L with the MUC1 oncoprotein 

**DOI:** 10.6026/97320630016937

**Published:** 2020-11-30

**Authors:** Ramajayam Govindan, Venkatachalam Sivabalan, Shazia Fathima JH, Umaphathy Vidhya Rekha, Senthilkumar Kalimuthu, Selvaraj Jayaraman, Kirubhanand Chandrasekaran

**Affiliations:** 1 Multidisciplinary Research Unit, Madurai Medical College, Madurai- 625 020, Tamil Nadu, India; 2 Department of Biochemistry, KSR Dental Sciences and Research, Thiruchengodu-637215, Tamil Nadu, India; 3 Department of Oral and Maxillofacial Pathology, Ragas Dental College and Hospitals, Chennai, India; 4 Department of Public Health Dentistry, Sree Balaji Dental College and Hospital, Pallikaranai, Chennai-600 100, India; 5 Central Research Laboratory, Melmaruvathur Adhiparasakthi Institute of Medical Sciences and Research, Melmaruvathur-603319, Tamil Nadu, India; 6 Department of Biochemistry, Saveetha dental college and Hospitals, Saveetha Institute of Medical and Technical Sciences, Saveetha University, Chennai-600 077, India; 7 Department of Anatomy, All India Institute of Medical Sciences, Nagpur, India

**Keywords:** Oral cancer, MUC1 protein, Justica adhatoda L, Molecular docking

## Abstract

The MUC1 oncoprotein is known to be linked with different types of cancer. Therefore, it is of interest to document the molecular docking analysis of compounds from Justica adhatoda L with the MUC1 oncoprotein. We report the structure based molecular binding features compounds such as amrinone, ethambutol, pyrazinamide and vasicoline the MUC1 oncoprotein for further consideration in drug discovery.

## Background

Early diagnosis of progression of cancer remains a challenge due to lack of early prognosis markers [1,2]. Mucins are high molecular weight glycoproteins, which play a key role in cell development, differentiation and cell signaling. The expression of the mucin gene is strongest in the respiratory, digestive and reproductive systems [3,4]. Cancer cells use mucin for cell proliferation, development, invasion, metastatic growth and defence towards innate immunity [5]. Over-expression of MUC1 is necessary to induce independent growth and tumorigenicity of the anchorage. The overexpression of MUC1 often confers tolerance to stress-induced cell death due to exposure to some genotoxic anticancer agents. The MUC1 over expression is conferred, at least to some extent, by the control of the MUC1 mRNA level at the transcriptional level. MUC1 communicates with ER and some other transcription factors, leading to the regulation of gene expression [6]. High expression of MUC1 is directly connected with tumour progression and metastasis, resulting in poor prognosis. In addition to the activity of mucins in the mechanical and chemical defence of cells, signal transduction could also be mediated by beta-catenin and MAP kinase, contributing in some cases to more violent tumour activity [7]. The expression levels of MUC1 in various human cancers have illustrated its function in cancer pathogenesis [8]. Therefore, it is of interest to document the molecular docking analysis of compounds from Justica adhatoda L with the MUC1 oncoprotein.

## Materials and Methods:

### Preparation of protein structure: 

The 3D coordinates of the crystal structure of MUC1 (PDB ID: 2ACM)[9] have been retrieved from the Protein Data Bank (http:/www.rcsb.org/pdb/home.do). MUC1 (chainA) was chosen for docking simulations.

### Ligand Preparation:

The 12-compounds of Justica adhatoda L have been collected from the PubChem compound database. It was prepared with the ChemBioDraw and the MOL SDF format of this ligand was converted into a PDBQT file using the PyRx method for generating atomic coordinates.

### Molecular docking studies:

Molecular docking study was conducted using AutoDock Vina in The Python Prescription (PyRx) 0.8 virtual screening tool [10]. The grid points in the X, Y and Z axes have been set. The grid core was positioned in the pocket core of the binding site. Protein and ligands have been translated to pdb.qt formats. Default docking algorithms have been set in accordance with the appropriate docking protocol. Individual docking procedures have been performed for each ligand protein complex. The findings have been ranked in the order of rising docking energies. The lowest binding energy of each cluster was considered representative [11]. Docked complexes were further anylysised by using PYMOL visualization [12].

## Results and Discussion:

**Figure 1:**
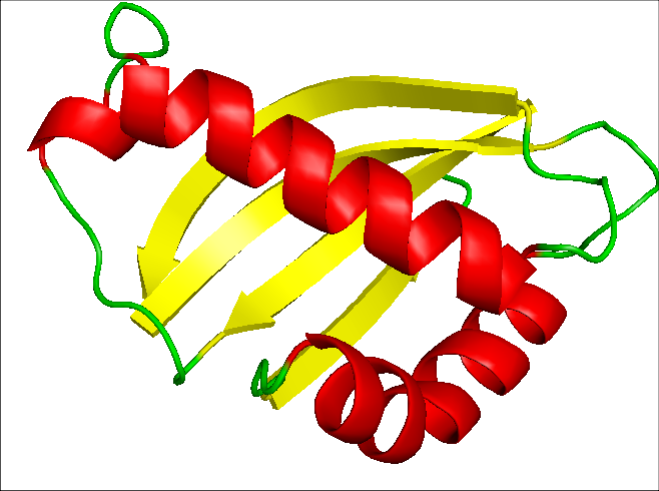
Structure of MUC1

**Figure 2:**
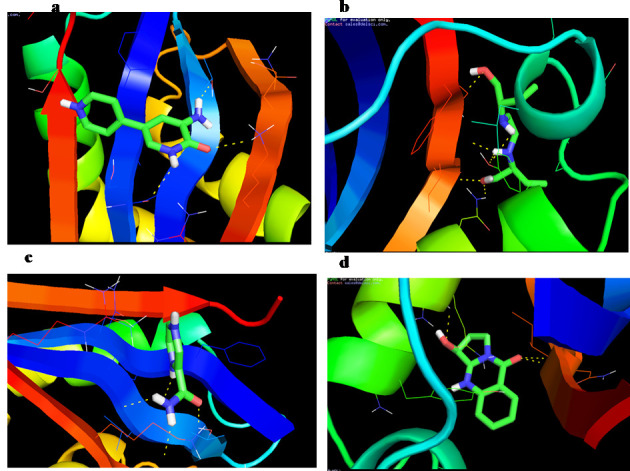
Molecular interaction of MUC1 with a) Amrinone; b) Ethambutol; c) Pyrazinamide; d) Vasicoline.

The biological activity of Justica adhatoda L compounds towards MUC1 was analyzed using the 3D structure of the receptor recovered from the protein data bank. For bioactive compound and proteins, a docked binding mode has been developed to connect the docking score method. The docking findings have been summarized in Table 1(see PDF). Further interaction research was carried out on ligands with binding affinity above four. The conclusions of the results have been purely based on the importance of the docking energy and the interaction at the binding sites. The more negative the value, the more reliable the complex and the more binding the affinity. As per energy funnel theory, less energy reflects extremely stable conformation. As a result, more energy is required to split the structure, which implies high-energy dissociation. The docking scores have been collected and shown in Table 2 (see PDF). The docking score was the highest for Amrinone with docking score -5.4 kcal / mol followed by Vasicoline, Ethambutol & Pyrazinamide with-5.1 kcal / mol,- 4.9 kcal / mol and 4.1 kcal / mol respectively. The structure of MUC1 was shown in Figure 1. An analysis of the binding pattern between the MUC1 protein and the ligands indicated which the binding pattern differed with the ligand type. The effects of the docking of the bioactive compounds from Justica adhatoda L have been shown in Figure 2. In order to analyse the relationship between the compounds and MUC1, the docked complexes were visualised using Pymol software. Out of the twelve docked complexes, we picked the best four complexes (Amrinone, Ethambutol, Pyrazinamide & Vasicoline) based on their score parameters and hydrogen bond interaction. All these four complexes formed the hydrogen bond interaction through the amino acids residues LYS-1093, GLN-1102, THR-1104, TYR-1066, GLN-1070, ILE-1092,PHE-1094, ASN-1091,GLN-1102,LEU-1103,THR-1104, ARG-1071,ASN-1091 & ILE-1092. Hence, these residues may be responsible functional amino acids of the protein MUC1. So inhibition of these residues with bioactive compounds was used to suppress the function of MUC1 protein. The results showed that all bioactive compounds with the target protein developed high negative e-values. It is also clear that bioactive compounds have been able to interact effectively with some of the available binding sites of the MUC1. Abovementioned study clearly shows that the bioactive compounds of Justica adhatoda L have been capable of inhibiting the function of the protein MUC1.

## Conclusion

We report the structure based molecular binding features compounds such as amrinone, ethambutol, pyrazinamide and vasicoline the MUC1 oncoprotein for further consideration in drug discovery.
